# Immunosuppression of the Trimellitic Anhydride-Induced Th2 Response by Novel Nonanatural Products Mixture in Mice

**DOI:** 10.1155/2013/748123

**Published:** 2013-11-19

**Authors:** Min-Jung Bae, Hee Soon Shin, Dong-Hwa Shon

**Affiliations:** ^1^Korea Food Research Institute, 1201-62 Anyangpangyo-ro, Bundang-gu, Seognam-si, Kyeonggi-do 463-746, Republic of Korea; ^2^Institute for Basic Science, School of Biological Sciences, Seoul National University, 599 Gwanak-ro, Gwanak-gu, Seoul 151-742, Republic of Korea

## Abstract

Many natural dietary products prevent or cure allergic inflammation; however, the ability of mixtures of these natural medicinals to suppress allergic skin inflammation is unknown. We examined the inhibitory effects of nonanatural products mixture (NPM-9), which provides immunoregulatory activation, on Th2-mediated skin allergic inflammation. Oral administration of NPM-9 in mice reduced ear thickness and specific IgE production in trimellitic anhydride- (TMA-)induced contact hypersensitivity (CHS). NPM-9 also suppressed IL-4 and IL-1*β* production in splenocytes but prevented only TMA-induced IL-1*β* production in inflamed ears. To characterize the mechanism of this effect, we examined NPM-9 immunosuppression on an OVA-induced Th2 allergic state. Oral administration of NPM-9 inhibited Th2-mediated serum IgE overproduction. NPM-9 also downregulated the polarized Th2 response, whereas it upregulated Th1 response in splenocytes. These data suggest that NPM-9 may be a useful therapeutic agent for allergic inflammatory diseases through its suppression of the Th2-mediated allergic response.

## 1. Introduction

Allergic dermatitis (AD) is characterized by allergic skin inflammation. Among the various types of AD, contact dermatitis is induced by an allergic response to a multitude of chemical substances associated with environmental contamination. Dysregulated type 1 helper T cell (Th1) and Th2 responses are pathogenic in allergic dermatitis via Th2 production of IL-4, IL-5, and IL-13 [1, 2]. Th2 cytokines promote mast cell development. Mast cells are key effectors in immunoglobulin (Ig) E-associated Th2-type immune responses because they are activated by cross linking of Fc*ε*RI. Allergen-provoked mast cells trigger release of allergic inflammatory mediators including histamine via degranulation [3]. IFN-*γ* suppresses IgE production by B cells, as well as IL-4 production from Th2 cells [4]. Therefore, the development of dermatitis is thought to be caused primarily by overproduction of Th2-mediated cytokines [5, 6].

Many therapeutic trials have evaluated agents that may modulate dermatitis, but prolonged use of these compounds causes a variety of side effects. Natural herbs, with their improved safety profiles and immune-regulatory effects, have been suggested as alternative therapeutics for the treatment of dermatitis and have been the subject of many studies [7–9]. It is unclear, however, whether natural products function synergistically to produce antiallergic effects on Th2 differentiation-induced contact dermatitis.

**Figure 1:**
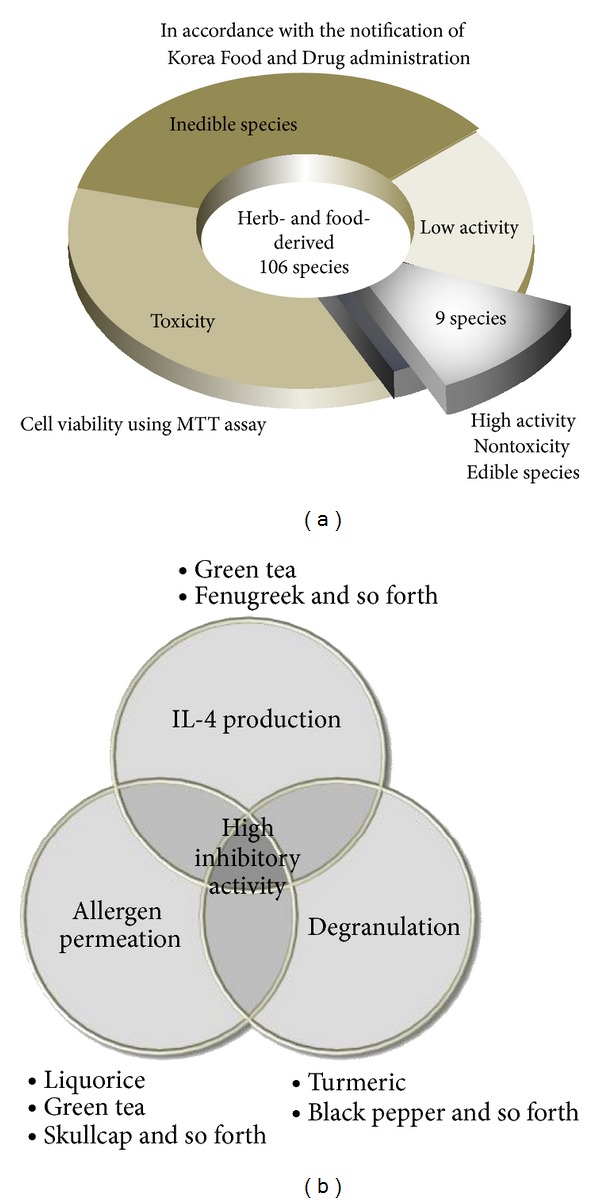
Flowchart representing selected criteria for NPM-9 from food- and herb-derived extracts. (a) The selection of food- and herb-derived species (edible, highly active, and nontoxic). (b) Selected food- and herb-derived species have a potent inhibitory activity against allergen permeation of human epithelial cells, IL-4 production in splenocyte T cells, and degranulation in mast cells.

We previously investigated the anti-allergic effects of natural product extracts derived from herbs and foods using various methods such as allergen permeation, Th2-related cytokines, and mast cell degranulation (Figure 1). We identified 4 food-derived extracts with high anti-allergenic potential, including black pepper (*Piper nigrum*), green tea (*Camellia sinensis*), turmeric (*Curcuma longa*), and fermented soybean paste (cheonggukjang), as well as herb-derived extracts of licorice (*Glycyrrhiza uralensis*), hawkweed (*Hieracium albiflorum*), beefsteak plant (*Perilla frutescens*), fenugreek (*Trigonella foenum-graecum*), and skullcap (*Scutellariae baicalensis*) (Table 1). Their curative properties have been demonstrated in many studies. Licorice, hawkweed, and skullcap suppress inflammation by regulating immune function in antigen-activated macrophages [10–12]. Beefsteak plant and green tea inhibit mast cell activation by suppressing histamine release and inflammatory cytokine and chemokine production [13–15]. Fenugreek, black pepper, and fermented soybean paste extracts and major constituents may serve as immunoregulatory mediators [16–19]. Turmeric produces an immunomodulatory response by stimulating dendritic cell function [20].

These 9 extracts were mixed to produce an effective treatment for allergic disorders after verifying their efficacy by assessing allergen permeation into the intestinal epithelium, Th2-related cytokine production in splenocytes, and mast cell degranulation. The mixture was named nona natural product mixture (NPM-9) and has been patented in Korea (Patent number 10-1141191).

The aim of this study was to investigate the anti-allergic effect of NPM-9 in trimellitic anhydride- (TMA-) induced contact hypersensitive (CHS) mice.

## 2. Materials and Methods

### 2.1. Animals

Female BALB/c mice, weighing 18 to 20 g, were purchased from OrientBio Inc. (Kyeonggi, Korea). The 4-week-old mice were housed in an air-conditioned room (23 ± 2°C) with a 12 h light/dark cycle. They were allowed free access to food and tap water. All animal experiments were performed according to the guidelines for animal use and care at the Korea Food Research Institute.

### 2.2. Sample Preparation

Herb- and food-derived elements were purchased from Kyungdong Oriental Medicine Market (Seoul, Korea) or a local market (Kyeonggi, Korea). Herbals were identified by Professor Y. Bu, Department of Herbal Pharmacology, Kyung Hee University. The specimens have been maintained by the functional materials research group, Korea Food Research Institute. Samples of each product (100 g) were reflux extracted twice in 1 L 70% ethanol using a Soxwave100 apparatus. Ethanol extracts were dried under a vacuum in a rotary evaporator. Concentrated extracts were lyophilized, yielding a dried powder that was stored at 4°C. The yield (%) of each product is provided in Table 2. Dried ethanol extracts were dissolved in saline (Sigma-Aldrich, St. Louis, MO) prior to use. NPM-9 (250 mg/kg) was mixed with the same volume of herbal (licorice, hawkweed, beefsteak plant, fenugreek, and skullcap) and food extracts (black pepper, green tea, turmeric).

### 2.3. Schedules for Mouse Sensitization, TMA Challenges, and Sample Treatment

**Figure 2:**
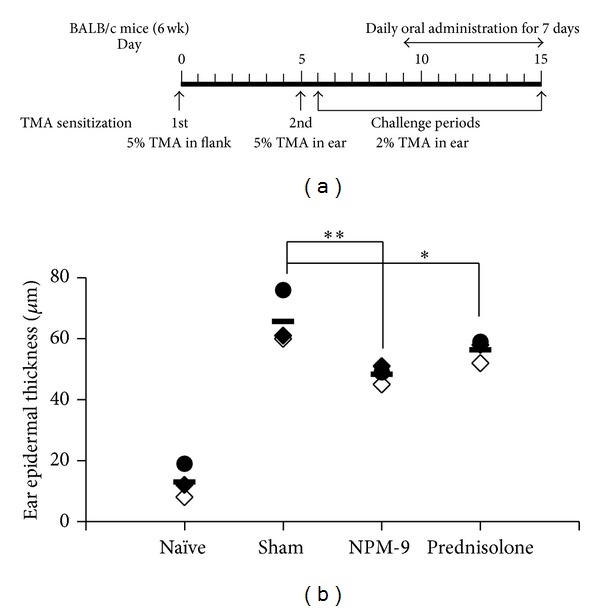
Experimental protocol and ear thickness in TMA-induced CHS mouse model. (a) Experimental protocol. (b) BALB/c mice were divided into naïve, sham (TMA), NPM-9 (250 mg/kg BW), and prednisolone groups (30 mg/kg BW). Ear epidermal thickness was measured by histological analysis (H&E staining). Values are presented as mean ± SD (*n* = 4 in each group). Data were analyzed by ANOVA followed by Student's *t* test. *P < 0.05, **P < 0.01, significantly different from the saline value.

TMA induction of CHS was performed as described in [21]. A schematic of the experimental procedure is shown in Figure 2(a). To induce CHS, mice were divided into naïve, sham, NPM-9, and prednisolone groups. Mice were sensitized with 50 *μ*L of 5% TMA (Sigma-Aldrich, St. Louis, MO) in solvent on shaved flank skin on day 0. Challenges with 10 *μ*L of 5% TMA in solvent on the dorsum of both ears were performed on day 5. In the chronic model, animals received challenges on the ears with 10 *μ*L of 2% TMA in solvent on days 6–15. A solvent control group was exposed to acetone and isopropyl myristate (4 : 1, v/v) throughout the duration of the experiment. In the treatment groups, NPM-9 (250 mg/kg body weight (BW)) and prednisolone (30 mg/kg BW), which served as a positive control, were administered orally 1 h before challenge. Each treatment was performed on days 9–15. Body weight and water intake were measured daily. Ear thickness was determined with a custom-built micrometer (Schering AG, Germany). Ears were mechanically homogenized in 2 mL PBS (Sigma-Aldrich, St. Louis, MO), centrifuged at 25,000 g for 30 min at room temperature, and cultured in the presence of 5 *μ*g/mL ConA. Cytokines were measured in the supernatant. Blood samples were obtained from the brachial plexus to estimate the immunoglobulin titer by ELISA 24 h after the final treatment. Spleens were removed and incubated with 5 *μ*g/mL ConA for 72 h. Cytokine production was measured by ELISA.

### 2.4. Sensitization with OVA and Preparation of Splenocyte Cultures

Mice were sensitized with 20 *μ*g OVA (Grade VI; Sigma-Aldrich, St. Louis, MO) adsorbed in 2 mg/mL Imject Alum (Pierce, Rockford, USA) and administered by intraperitoneal (i.p.) injection on days 7 and 14. Splenocytes were prepared by aseptically removing the spleen from each mouse. Homogenized single spleen cells were collected and treated with red blood cell- (RBC-) lysing buffer (Sigma-Aldrich). The splenocytes were adjusted to 5 × 10^6^ cells/mL in RPMI medium using the trypan blue dye exclusion method. The splenocytes (200 *μ*L/well) were then cultured in the presence or absence of OVA (100 *μ*g/mL/well) and each herb and food extract. The plates were incubated at 37°C for 72 h in a humidified incubator with 5% (v/v) CO_2_ and 95% (v/v) air. Cytokines in the supernatant were measured by ELISA.

### 2.5. Oral NPM-9 Treatment in OVA-Sensitized Mice

To investigate NPM-9 inhibition of the OVA-induced allergic response *in vivo*, mice were divided into normal (naïve), untreated (sham), and NPM-9-treated groups. Mice were sensitized with 20 *μ*g OVA adsorbed in 2 mg/mL Imject Alum i.p. injection on days 7 and 21. The solvent control group was administered saline and 2.5% ethanol throughout the experiment. In the treatment groups, 250 mg/kg NPM-9 was administered orally. NPM-9 treatments were performed on days 14–28. A schematic of the experimental procedure is shown in Figure 4(a). Twenty-four hours after the last treatment, blood samples were obtained from the brachial plexus. Splenocytes were prepared from each mouse. 

### 2.6. Measurement of Serum IgE

IgE antibody levels in sera were measured by ELISA. Aliquots (200 *μ*L per well) of IgE capture antibody (BD PharMingen, San Diego, CA, USA) or OVA (10 *μ*g/mL dissolved in 0.1 mol/L NaHCO_3_ (Wako Pure Chemical Industries), pH 8.2) were pipetted into 96-microwell plates (Nunc, Thermo Fisher Scientific, Roskilde, Denmark). The plates were incubated overnight at 4°C and then carefully washed 3 times with washing buffer (0.5 g/L Tween 20 in PBS). Serum samples were diluted 1 : 5 for specific IgE determinations. Aliquots (100 *μ*L) of diluted serum samples were added to the wells. Pooled sera from nonssensitized and sensitized mice were included as negative and positive controls. The levels were determined using biotin-conjugated rat anti-mouse IgE (BD PharMingen, San Diego, CA, USA) according to manufacturer protocols. The plates were read using an ELISA plate reader (Molecular Devices, Inc.) at 450 nm.

### 2.7. Measurement of Cytokine Levels Using ELISA

A cytokine assay kit (BD PharMingen, San Diego, CA, USA) was used to measure cytokine levels (IFN-*γ*, IL-12, IL-4, and IL-10), according to manufacturer protocols. Briefly, supernatants and standard solution were transferred to 96-well plates precoated with monoclonal antibodies to each of the target cytokines and then incubated at room temperature for 2 h. After thorough washing with the washing buffer included in the kit, a horseradish peroxidase- (HRP-) conjugated secondary antibody was added to each well, and incubation was continued at room temperature for 2 h. After removal of the secondary antibody, the substrate solution for the enzymatic reaction was added, and samples were incubated for another 30 min in the dark. The reaction was terminated by addition of stop solution, and absorbance was measured at 450 nm in a microplate reader (Molecular Devices, Inc.). The IC_50_ value of IL-4 was calculated from the reduction of IL-4 by different concentrations of test substance using linear regression analysis.

### 2.8. Statistical Analysis

Each result is expressed as the mean ± SD. Differences were assessed by ANOVA followed by Student's *t*-test.

## 3. Results and Discussion

### 3.1. Oral Administration of NPM-9 Attenuates TMA-Induced Infiltration of Inflammatory Cells in the Ear Dermis

In the BALB/c model of TMA-induced CHS, mice are sensitized on the flank skin, and T-cell-dependent inflammation of the ear skin is induced by topical challenges with TMA (Figure 2(a)). The TMA-induced response increased production of Th2 cytokines and immune cell infiltration, such as by mast cells [21]. The severity of inflammation can be assessed using ear thickness [22]. In all but the naïve group, 5% TMA challenge on days 0 and 5 and subsequent daily low-dose challenges with 2% TMA for 10 days induced a significant increase in ear thickness; this increase was inhibited by prednisolone (30 mg/kg BW), which served as the positive control [18]. Daily NPM-9 gavage (250 mg/kg BW) also produced an incremental decline in ear thickness (Figure 2(b)). We also measured epidermal thickness, infiltration of eosinophils and lymphocytes, and the number of mast cells in the dermis of the ears and found that NPM-9 inhibited the effects of TMA (data not shown). We also observed the body weight of each mouse in days 5~20 on Th2-mediated skin allergic inflammation to examine toxicity or side effect of NPM-9. As a result, body weight of NPM-9 group did not differ from that of sham or prednisolone group (data not shown).

### 3.2. Oral Administration of NPM-9 Suppressed IgE Levels in TMA-Induced CHS Mice

**Figure 3:**
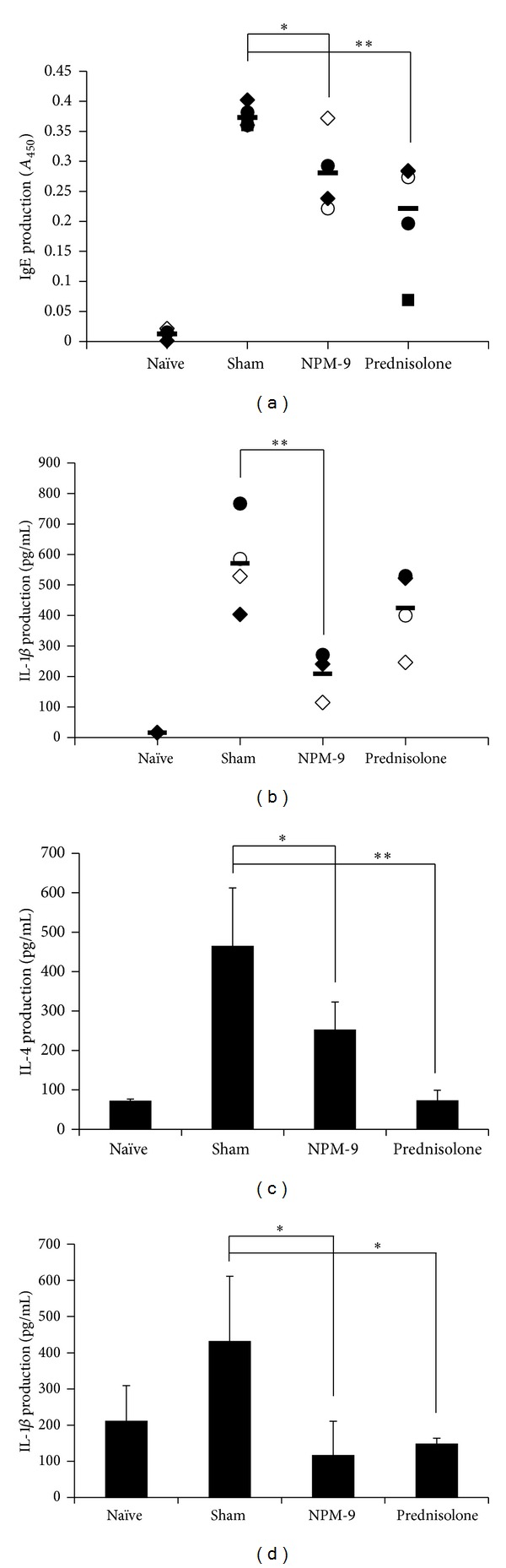
Inhibitory effect of NPM-9 in inflamed regions and splenocytes from TMA-induced allergic mice. (a) IgE levels in serum were measured by ELISA. To measure IgE, collected sera were diluted 1 : 50. (b) IL-1*β* was quantified by ELISA after homogenization in PBS-T using inflamed ear skin from TMA-induced BALB/c mice. Secreted (c) IL-4 and (d) IL-1*β* were quantified by ELISA after 72 h culture using splenocytes from TMA-induced BALB/c mice. Values are presented as mean ± SD (*n* = 4 per group). Data were analyzed by ANOVA followed by Student's *t* test. *P < 0.05, **P < 0.01, significantly different from the saline value.

TMA-sensitized and -challenged mice received orally administered NPM-9 (250 mg/kg) daily for 14 days. Serum was collected from each group for serum IgE measurements (Figure 3(a)). The TMA-induced CHS model displays characteristics of AD such as increased IgE. Allergen-specific IgE triggers local inflammatory responses, eventually generating various allergic responses [23]. In this study, serum IgE levels in TMA-treated CHS mice were higher than in nontreated mice, and oral NPM-9 administration suppressed this effect (Figure 3(a)). The capability of NPM-9 to reduce serum TMA-specific IgE was compared to that of prednisolone.

### 3.3. NPM-9 Administration Suppresses TMA-Induced Cytokine Expression in Ear Tissue and Splenocytes

We examined whether NPM-9 inhibits the production of Th2 cytokine IL-4 and inflammatory cytokine IL-1*β* in inflamed ear tissue and splenocytes. Oral administration of NPM-9 reduced the level of inflammatory cytokine IL-1*β* in inflamed ear tissue but not IL-4 (Figure 3(b)). In splenocytes, NPM-9 reduced IL-4 and IL-1*β* without cytotoxicity (Figures 3(c) and 3(d)). It is possible that NPM-9 had not yet induced Th2 migration to the inflamed regions but was sufficiently potent to downregulate Th2 in splenocytes. These results show that oral administration of NPM-9 constricted TMA-induced dermal inflammation; the biological basis of this inhibition appears to involve the balance of Th2 cells in the immune system. It is also important to demonstrate IFN-*γ* production in skin inflammation. There are distinct types of TMA-induced skin inflammation mouse models: acute TMA-induced CHS in Balb/c mice with subacute and chronic models of TMA-induced ear inflammation. In comparison to the acute model, the chronic TMA-induced CHS model exhibits a mixed type 1 and 2 T-cell differentiation and activation pattern [21]; however, the TMA-induced CHS mouse model used in this study is acute or subacute, characterized by eosinophil and T-cell infiltration, Th2 cytokine production, and IgE expression [21]. Moreover, oral administration of NPM-9 suppressed early-phase Th2 skewing, prohibiting the development of chronic inflammation. Therefore, we examined the Th2-dependent immune responses (IgE and IL-4) and proinflammatory cytokine IL-1*β* as indicators of Th2-mediated skin inflammation; however, the effect of NPM-9 on IFN-*γ* production in both Th1-s and Th2-dependent chronic skin inflammation requires further study. 

### 3.4. Synergistic Inhibition of IL-4 Production in *Ex Vivo* Splenocytes from OVA-Sensitized BALB/c Mice

To determine how skin inflammation is inhibited by suppression of skewing to Th2 cells, we examined how each extract inhibited Th2 polarization* ex vivo*. Levels of IL-4 in splenocytes were determined using the OVA-induced Th2 allergic mouse as an *ex vivo* model. Inhibition of Th2 polarization was measured as the IC_50_ with IL-4. IC_50_ values were calculated and compared as illustrated in Table 3. IL-4 production was inhibited by fenugreek > black pepper > chonggugjang > licorice > green tea > skullcap > turmeric > hawkweed > beefsteak plant. Each extract produced an antiallergic effect by inhibiting IL-4 production; food extracts were more effective than herb extracts. Splenocytes cultured with NPM-9 produced IL-4 levels 1.6- to 16-fold lower than that with each extract alone (Table 3). The IC_50_ ratio of IL-4 versus IFN-*γ* in the presence of NPM-9 was 1.6- to 48-fold higher than that with each extract alone. These results indicate that NPM-9 exerts potent synergistic suppression of the allergic response by inhibiting IL-4 secretion and inducing IFN-*γ* secretion.

### 3.5. Oral Administration of NPM-9 Suppressed the Serum IgE in OVA-Induced Allergic Mice

**Figure 4:**
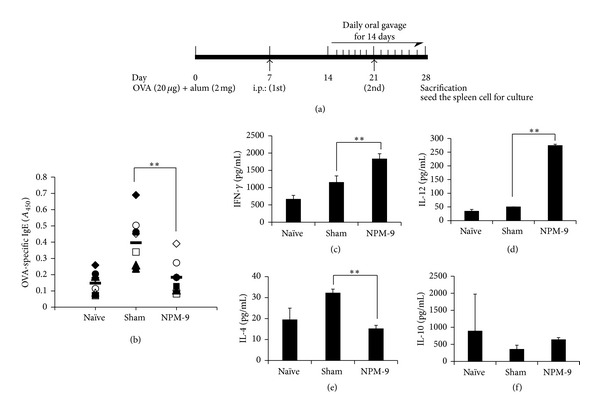
NPM-9 suppression of OVA-specific IgE and IL-4 production in OVA-sensitized BALB/c mice. (a) Experimental protocol for OVA-induced allergic response in mice. (b) OVA-specific IgE levels in serum were measured by ELISA. To determine total and OVA-specific antibody titers, the collected sera were diluted 1 : 50 for IgE detection. Secreted (c) IFN-*γ*, (d) IL-12, (e) IL-4, and (f) IL-10 were quantified by ELISA after 72 h culture using splenocytes from OVA-sensitized BALB/c mice. Values are presented as mean ± SD (*n* = 7 per group). Data were analyzed by ANOVA followed by Student's *t* test. *P < 0.05, **P < 0.01, significantly different from the saline value.

We also examined how oral administration of NPM-9 inhibits the OVA-induced Th2-mediated allergic response. OVA-sensitized mice received orally administered NPM-9 (250 mg/kg) daily for 14 days (Figure 4(a)). Serum from each group was collected for measurement of OVA-specific IgE (Figure 4(b)). Th2 cells play an important role in the OVA-induced mouse model by their influence on IL-4 and IL-5 production [24]. IL-4 is the major inducer of class switching of Ig to B lymphocyte IgE biosynthesis associated with allergic responses [23]. In this study, NPM-9-treated mice exhibited a significant reduction in serum OVA-specific IgE than nontreated mice in the Th2-mediated allergic state.

### 3.6. Oral Administration of NPM-9 Inhibits the Production of Th2 Cytokines in Splenocytes from OVA-Induced Th2-Mediated Allergic Mice

To determine how NPM-9 administration in OVA-sensitized mice modulates the Th1 and Th2 pathways, splenocyte levels of IL-4 and IFN-*γ* were determined using the OVA-induced allergic mouse described above. IFN-*γ* and IL-12 levels were greater in the splenocytes of NPM-9-treated OVA-stimulated mice than in untreated mice. IL-4 was suppressed in the splenocytes of NPM-9-treated versus the untreated OVA-stimulated group. However, IL-10 secretion was not enhanced by NPM-9 treatment (Figures 4(c)–4(f)). The effect of NPM-9 on IL-12 and IL-10 is interesting in the context of macrophages. IL-12, a product of activated macrophages, induces the Th1 cytokine pattern [25], and IL-10 inhibits macrophage-dependent cytokine synthesis by Th1 cells [26]. That is, Th1 cells can be inhibited by IL-10 but induced by IL-12 through the regulation of macrophages. Consistent with this model, we speculated that NPM-9 suppression of Th2-mediated allergic responses such as skin inflammation may occur through the induction of Th1 via macrophages. 

Based on the immunomodulatory effects of NPM-9 in the TMA-induced dermatitis model, we conclude that the diverse active compounds of NPM-9 have synergistic effects on allergic responses. The main chemical constituents in each compound include coumarin, luteolin, and caffeic and chlorogenic acids in hawkweed [27]; rosmarinic acid in beefsteak plant [28]; baicalein and wogonin in skullcap [29]; glycyrrhizin in licorice [30]; poly *γ*-glutamic acid and isoflavone in fermented soybean paste (Cheonggukjang) [13]; alkylamides and piperine in black pepper [12]; saponin and flavonoid in fenugreek [31]; curcumin in turmeric [14]; and catechin in green tea [32]. Many studies have reported that flavonoids such as curcumin or catechin have immunomodulatory activities. Glycyrrhizin enhances LPS-induced IL-12 production by peritoneal macrophages independent of IFN-*γ* and GM-CSF [33]. Saponin and poly *γ*-glutamic acid have immunostimulatory effects on the immune function of lymphocytes or the immune responses to vaccine immunizations [14, 34, 35]. Oral administration of NPM-9 (250 mg/kg/d) for 2 weeks had no adverse effects in mice, demonstrating that NPM-9 is safe even at high concentrations. The safety and immunomodulatory effects of NPM-9 may result from the interaction of active ingredient in each extract. Thus, NPM-9 may be a safe, natural therapy for allergic diseases such as atopic dermatitis, allergic coryza, and asthma.

## 4. Conclusion

We have demonstrated that NPM-9 suppresses allergic skin inflammation in a TMA-induced CHS mouse model and exerts its effect by inhibiting a skewed Th2 response. NPM-9 inhibits the polarized Th2 response through Th1 skewing that is dependent on IFN-*γ* and IL-12 secretion in an OVA-induced Th2 allergic state. Our results provide scientific proof of the prevention and treatment of skin allergic inflammation by the synergistically inhibitory effects of food and herb extracts on Th2-polarized allergic responses. 

## Figures and Tables

**Table 1:** The activity of food- and herb-derived species.

Sample extract	Scientific name	Division	Relative activity		
			Inhibitory activity on allergen permeation	Inhibitory activity on IL-4 production	Inhibitory activity on degranulation
Licorice	*Glycyrrhiza uralensis *	Herb	+++	+	+
Hawkweed	*Hieracium albiflorum *	Herb	++	++	++
Beefsteak plant	*Perilla frutescens *	Herb	++	++	+
Fenugreek	*Trigonella foenum-graecum *	Herb	++	+++	++
Skullcap	*Scutellariae baicalensis *	Herb	+++	++	++
Black pepper	*Piper nigrum *	Food		++	+++
Green tea	*Camellia sinensis *	Food	+++	+++	++
Turmeric	*Curcuma longa *	Food	+	+	+++
Fermented soybeans paste (cheonggukjang)		Food	++	++	++

+++: very strong; ++: strong; +: mild.

**Table 2:** Extraction yield for food- and herb-derived species.

Extraction yield (w/w, %)								
Licorice	Hawkweed	Beefsteak plant	Fenugreek	Skullcap	Black pepper	Green tea	Turmeric	Fermented soybeans paste (cheonggukjang)
21.15	20.22	45.68	17.50	13.50	33.08	30.27	48.47	19.69

**Table 3:** IC_50_ of NPM-9 for IL-4 and IFN-*γ*.

Sample extract	IC_50_ for IL-4 (ug/mL)	Ratio of IFN-*γ*/IL-4
Licorice	62.7	3.00
Hawkweed	208	0.15
Beefsteak plant	213	0.10
Fenugreek	15.4	2.50
Skullcap	86.7	0.98
Black pepper	31.3	1.45
Green tea	80.3	0.23
Turmeric	148	0.15
Fermented soybeans paste (cheonggukjang)	54.3	0.82
NPM-9	12.7	4.80

IC_50_ of IFN-*γ* and IL-4 were quantified by ELISA after cells were cultured for 72 h with splenocytes from OVA-induced allergic mice in the presence of each extract or NPM-9, Natural Nonaproduct Mixture.

## Abbreviations

AD: Allergic dermatitis
APC: Antigen-presenting cells
CHS: Contact hypersensitivity
DNP: Dinitrophenyl
ELISA: Enzyme-linked immunosorbent assay
HRP: Horseradish peroxidase
IFN: Interferon
Ig: Immunoglobulin
IL: Interleukin
NPM-9: Nonanatural products mixture
OVA: Ovalbumin
Th: T-helper
TMA: Trimellitic anhydride.
